# Aerobic pathogen profiles and antibiotic resistance in diabetic foot ulcers: Data from a Specialized Diabetes Center in Jordan

**DOI:** 10.1097/MD.0000000000047908

**Published:** 2026-03-06

**Authors:** Dana Hyassat, Mohammad Al Gazo, Faris Bakri, Yousef Khader, Laith Baqain, Amro Al-Simadi, Eid Samara, Mohammad Al Masri, Jood Aladwan, Shiraz Mheidat, Dalia Hanash, Jamal Wadi Al Ramahi, Mohammad El-Khateeb, Kamel Ajlouni

**Affiliations:** a The National Center for Diabetes, Endocrinology, and Genetics, The University of Jordan, Amman, Jordan; b Division of Infectious Diseases, Department of Medicine, Jordan University Hospital, Amman, Jordan; c Infectious Diseases and Vaccine Center, University of Jordan, Amman, Jordan; d Jordan University of Science and Technology (JUST), Irbid, Jordan; e School of Medicine, University College Dublin, Dublin, Ireland; f Adjunct Faculty, Jordan University Hospital, Amman, Jordan.

**Keywords:** diabetes, diabetic foot ulcer, methicillin-resistant *Staphylococcus aureus* (MRSA), multidrug resistance

## Abstract

This study aimed to identify the aerobic bacterial profile and antibiotic susceptibility patterns in patients with infected and uninfected diabetic foot ulcers (DFUs) at the National Center for Diabetes, Endocrinology, and Genetics (NCDEG), Amman, Jordan. A single-center, cross-sectional study was conducted from November 9, 2022 to January 2, 2023, involving 171 patients with DFUs. Superficial swabs and tissue specimens were collected, and antibiotic susceptibility testing was performed following Clinical and Laboratory Standards Institute (CLSI) guidelines. A total of 260 bacterial isolates were obtained, with an average of 1.6 pathogens per participant. Gram-positive bacteria (GPB) accounted for 65.1% of isolates, while Gram-negative bacteria represented 28.4%. The prevalence of monomicrobial and polymicrobial infections was nearly equal (50%). Among the 181 GPB isolates, *Staphylococcus aureus* was the most predominant pathogen, representing 39.8% of all GPB isolates. Notably, 68.1% of *S aureus* isolates were identified as methicillin-resistant *Staphylococcus aureus*. The GPB isolates exhibited complete susceptibility to vancomycin, linezolid, tigecycline, and teicoplanin. For Gram-negative bacteria, ertapenem, meropenem, amikacin, and imipenem were the most effective antibiotics. This study highlights the predominance of GPB, particularly *S aureus*, in DFUs, with a significant proportion of methicillin-resistant *Staphylococcus aureus* isolates. The findings underscore the importance of continued surveillance of bacterial profiles and antibiotic susceptibility patterns in DFUs. Future research is needed to guide the judicious use of antibiotics in empirical therapy for diabetic foot infections.

## 1. Introduction

Diabetic foot infections (DFIs) are a significant complication of diabetic foot ulcers (DFUs), affecting up to 82% of hospitalized cases and often resulting in severe outcomes, including lower limb amputations.^[1,2]^ Clinically, DFIs are characterized by the presence of inflammatory signs in any tissue below the malleoli, with infection confirmed by 2 or more of these signs. However, conditions such as peripheral neuropathy and vascular disease can mask symptoms, leading to delayed diagnosis and rapid progression of infections.^[3]^

Chronic ulcers often harbor diverse bacterial populations that influence the severity and duration of DFUs.^[4]^ Infections may be monomicrobial or polymicrobial, with severe DFIs typically involving multiple bacterial species.^[3]^ While Gram-positive cocci are the most commonly isolated pathogens, Gram-negative bacilli are also frequently identified.^[5]^

Previous studies in Jordan consistently show DFUs yield polymicrobial infections, often dominated by Gram-negative rods. In 1 southern-Jordan cohort, 45.3% of isolates were Gram-negative, chiefly *Escherichia coli* (17%), *Pseudomonas aeruginosa* (8%) and *Acinetobacter* spp. (11%).^[6]^ Common Gram-positive isolates included *Staphylococcus aureus* (~11%) and enterococci (~2%). Coagulase-negative staphylococci (CoNS; mainly *Staphylococcus epidermidis*) were also frequent: in an Amman-based series staphylococci appeared in 48% of DFUs (with *S aureus* in 14.2% of wounds). Yeasts (*Candida*, ~3%) and mixed cultures or contaminants were reported in a minority of cases.^[7]^

Diabetic foot infections impose substantial financial burdens and are associated with a 2.5-fold increase in mortality risk compared to patients without infections.^[8]^ Effective treatment hinges on appropriate antimicrobial selection, underscoring the importance of ongoing research into causative microorganisms and resistance patterns, particularly with the rise of multidrug-resistant organisms (MDROs).^[9]^ According to the Infectious Diseases Society of America (IDSA) guidelines, clinically infected DFUs should be treated with empirical antibiotics until culture results are available. These empirical regimens should provide broad-spectrum coverage for the most common pathogens but must be tailored based on local epidemiological data and culture results to minimize resistance development.^[10]^

The prevalence of MDROs, particularly methicillin-resistant *Staphylococcus aureus* (MRSA), in DFUs highlights the need for targeted empirical antibiotic therapy.^[11,12]^ Misdiagnosis or inappropriate antibiotic use can exacerbate pathogen virulence and contribute to drug resistance.^[13,14]^

This study aimed to identify the aerobic bacterial profile and antibiotic susceptibility patterns in patients with infected and uninfected DFUs attending the National Center for Diabetes, Endocrinology, and Genetics (NCEDG) in Amman, Jordan.

## 2. Materials and methods

### 2.1. Study design

This single-center cross-sectional study was conducted on patients with DFUs from November 9, 2022 to January 2, 2023. Eligible participants included all attending patients to the diabetic foot clinic and aged 18 years or older with DFUs located below the ankle. Exclusion criteria included nondiabetic ulcers, ulcers above the ankle, autoimmune diseases, pregnancy, or gangrene classified as Wagner grade 4 or 5. This study received approval from the NCDEG Ethics Committee on November 14, 2022 (IRB No. 1/2022). Written informed consent was obtained from all participants.

### 2.2. Sample size

To estimate the sample size for identifying antibiotic susceptibility patterns in patients with infected and uninfected DFUs, we used the standard formula for cross-sectional studies. We applied a confidence level of 95%, corresponding to a *Z* value of 1.96, and assumed an estimated prevalence of antibiotic resistance of 50% to maximize the sample size. The margin of error was set at 10% to maintain feasibility while ensuring reasonable precision. Based on these parameters, the calculated sample size was approximately 97 participants. However, the sample size was increased to 170 to enhance the study’s robustness and account for potential data variability.^[15]^

### 2.3. Data collection

Sociodemographic and relevant data, including age, sex, education level, occupation, and smoking status, were collected through face-to-face interviews. Clinical data such as diabetes mellitus duration, ulcer duration, and current antibiotic use were also obtained. The size and location of the ulcers were assessed and measured in the clinic. Specimen collection methods were determined after a thorough clinical evaluation of the ulcers. Additional information, such as hemoglobin A1c (HbA1c) levels and body mass index (BMI), was extracted from medical records.

### 2.4. Specimen collection

Specimens were collected using aseptic techniques from patients with DFUs, regardless of antibiotic use, to study colonization in clinically uninfected DFUs. Clinically uninfected ulcers were sampled to detect the presence of bacterial colonizers that may not yet manifest as an active infection. This approach helps identify potential pathogens that could become clinically significant if the ulcer becomes infected. Therefore, while the study included uninfected DFUs, the focus remained on understanding the aerobic bacterial landscape, whether colonizing or infecting, to better inform empirical therapy and infection control. Before sampling, sharp debridement was performed to remove slough and necrotic tissue after cleaning the ulcer with sterile gauze and normal saline. Only 1 sample was collected from each participant, specifically from the most distal ulcer, to maintain a 1:1 participant-to-specimen ratio.

Grade 1 and 2 ulcers were sampled using sterile superficial swabs to avoid causing harm to the tissue bed. For grade 2 and 3 ulcers, either surface swabs or tissue cultures were collected, depending on the presence of nonviable tissue and the need for sharp debridement. Tissue samples were taken aseptically using sterile instruments during debridement.

Surface swabs were obtained following Levine’s Technique, which involves applying pressure to extract fluid from the ulcer bed while rotating the swab over a 1 cm^2^ area for 5 seconds.^[16]^ Samples were immediately sealed and transported to the microbiology laboratory at the NCDEG using Copan Liquide Amies Elution Swabs (ESwabs; Copan, Brescia, Italy), which preserve a wide range of microorganisms. Specimens were processed within 2 hours of collection or refrigerated if delayed.

### 2.5. Microorganism identification

Swab cultures were inoculated onto brain heart infusion agar (Mast, Bootle, Liverpool, UK) with 5% sheep red blood cells, cystine-lactose-electrolyte-deficient agar (Oxoid, Basingstoke, Hampshire, UK), and MacConkey agar (Oxoid, UK), and incubated aerobically at 35°C with 5% CO_2_ for up to 48 hours. Tissue cultures were initially inoculated in thioglycolate broth (Oxoid, UK) for 24 hours before being transferred to solid media. Bacterial growth was reported regardless of bacterial quantity.^[17,18]^ Anaerobic organisms were not investigated. Chromogenic agar (Biomaxima, Lublin, Poland) was used for specific bacterial differentiation.

### 2.6. Susceptibility testing

Antimicrobial susceptibility testing was performed using the Kirby–Bauer disc diffusion method, following Clinical and Laboratory Standards Institute (CLSI) guidelines (2022).^[17]^ Bacterial colonies were suspended in sterile normal saline and adjusted to match the 0.5 McFarland standard. Mueller–Hinton agar plates (Oxoid, UK) were inoculated uniformly with sterile swabs. After a brief drying period, antimicrobial disks were placed on the plates, which were incubated at 35°C for 18 to 20 hours. For *Haemophilus* species and *Streptococci*, plates were incubated in an environment with 5% CO_2_.^[18,19]^ A variety of antimicrobial disks were used, including Ampicillin, Levofloxacin, and Vancomycin, among others, all sourced from Oxoid, UK.^[17,19]^

### 2.7. Definition of variables

Diabetic foot ulcers were classified according to the Wagner–Meggitt grading system^[20]^:

- Grade 0: No ulcer.
- Grade 1: Full-thickness skin ulcer.
- Grade 2: Ulcer involving muscle, tendon, or joint capsule.
- Grade 3: Deep ulcer reaching bone or joint, with deep-seated abscess or osteomyelitis.
- Grade 4: Limited gangrene.
- Grade 5: Gangrene extending proximally to involve the entire foot.

Clinical suspicion of infection was based on guidelines from the International Working Group on the Diabetic Foot (IWGDF) and the Infectious Disease Society of America (IDSA)^[21]^:

- Grade 1 (uninfected): No purulence or other signs of inflammation.
- Grade 2 (mild): At least 2 signs of inflammation (e.g., purulence, tenderness, erythema, induration, or warmth). Any erythema surrounding the ulcer extends 2 cm or less, with the infection limited to skin or superficial subcutaneous tissues. No systemic signs or complications.
- Grade 3 (moderate): Infection as in grade 2, but with additional signs such as cellulitis extending beyond 2 cm, lymphangitic streaking, deep abscesses, gangrene, or spread beneath superficial fascia to involve muscle, tendon, joint, or bone. The patient is metabolically and systemically well.
- Grade 4 (severe): Infection associated with systemic toxicity or metabolic instability (e.g., fever, tachycardia, chills, hypotension, vomiting, confusion, acidosis, leukocytosis, azotemia, or severe hyperglycemia).

Multidrug-resistant (MDR), extensively drug-resistant (XDR), and pandrug-resistant (PDR) isolates were defined based on European Centre for Disease Prevention and Control (ECDC) and Centers for Disease Control and Prevention (CDC) criteria:

- MDR: Resistance to at least 1 agent in 3 or more antimicrobial categories.
- XDR: Resistance to all but 1 or 2 antimicrobial categories.
- PDR: Resistance to all agents in all antimicrobial categories.^[22]^

Controlled diabetes was defined as an HbA1c level of 7% or less, in accordance with the American Diabetes Association (ADA) guidelines.^[23]^ Obesity and overweight were defined based on BMI, which was calculated by dividing a person’s weight in kilograms by the square of their height in meters (kg/m^2^). A BMI between 18.5 and 24.9 was considered normal. Individuals with a BMI between 25 and 29.9 were classified as overweight, while those with a BMI of 30 or higher were categorized as obese.^[24]^

### 2.8. Statistical analysis

Data were analyzed using IBM SPSS Statistics, version 24 (IBM Corp., Armonk). Categorical variables were summarized as frequencies and percentages, while continuous variables were presented as means with standard deviations (SD). The chi-square test was used to assess associations between antibiotics and bacterial isolates classified as MDR, XDR, and PDR. Statistical significance was defined as a *P*-value of <.05.

## 3. Results

### 3.1. Patients’ characteristics

A total of 171 diabetic patients participated in this study, of whom 82% had type 2 diabetes and 18% had type 1 diabetes. From these patients, 278 bacterial isolates were identified, with an average of 1.6 pathogens per participant. Participants’ ages ranged from 18 to 83 years, with a mean (SD) age of 57.6 (11.7) years. The sample consisted of 64% males and 36% females. Among the participants, 49% were nonsmokers, while 36% were current smokers.

Approximately 50% of the participants were classified as obese, and 37% were overweight. Uncontrolled diabetes (HbA1c ≥ 7%) was observed in 81% of participants, while 19% had controlled diabetes (HbA1c < 7%). The duration of diabetes varied, with 37% of participants living with diabetes for <15 years, 27% for 15 to 20 years, and 36% for more than 20 years, with a mean duration of 18.1 (9.0) years. Sociodemographic and clinical characteristics are detailed in Table 1.

**Table 1:** Sociodemographic and clinical characteristics of the study population (N = 171).

Variables	n	%
Age (yr)		
<55	56	32.8
55–60	52	30.4
>60	63	36.8
Gender		
Male	109	63.7
Female	62	36.3
Education		
Illiterate	9	5.3
High school or less	113	66.1
Higher than high school	49	28.6
Occupation		
Employee	48	28.1
Unemployed	30	17.6
Retired	43	25.1
Housewife	50	29.2
Smoking		
Current smoker	61	35.7
Ex-smoker	26	15.2
Nonsmoker	84	49.1
Body mass index (kg/m^2^)		
Normal (18.5–24.9)	21	12.3
Overweight (25–29.9)	64	37.4
Obese (≥30)	86	50.3
Duration of diabetes (yr)		
<15	64	37.4
15–20	46	26.9
>20	61	35.7
Type of diabetes		
T1DM	30	17.5
T2DM	141	82
HBA1c (%)		
˂7	29	19.1
≥7	123	80.9

HbA1c = hemoglobin A1c, T1DM = type 1 diabetes mellitus, T2DM = type 2 diabetes mellitus.

### 3.2. Ulcer related characteristics

Table 2 shows ulcer related characteristics in 171 patients with diabetes. The duration of ulcers ranged from 1 day to 10 years, with a mean (SD) duration of 205.7 (510.5) days. Ulcer sizes varied between 0.3 and 22.4 cm^2^, with a mean (SD) size of 5.4 (22.0) cm^2^. The most common ulcer locations were the toes (40.9%), followed by the plantar aspect (39.2%) and the forefoot (29.8%). Based on the Wagner classification, grade 2 ulcers were the most frequent (42%), followed by grade 3 (37%) and grade 1 (21%).

**Table 2:** Ulcer related characteristics in 171 patients with diabetes.

Variables	n	%
Duration of ulcer (d)		
≤60	98	57.3
>60	73	42.7
Location of ulcer		
Right foot	77	45
Left foot	94	55
Planter	67	39.2
Dorsal	20	11.7
Forefoot	51	29.8
Midfoot	22	12.9
Hindfoot	18	10.5
Medial	16	9.4
Lateral	12	7
Toes	70	40.9
Lateral malleolus	5	2.9
Medial malleolus	2	1.2
Ulcer grade		
Grade 1	36	21.1
Grade 2	72	42.1
Grade 3	63	36.8
Infection grade		
No infection	67	39.2
Mild	17	9.9
Moderate	78	45.6
Severe	9	5.3
Specimens type		
Swab culture	149	87.1
Tissue culture	22	12.9

Clinically, 61% of ulcers exhibited signs of infection, while 39% were not infected. Among the clinically infected ulcers, 9.9% were classified as mild infections, 45.6% as moderate infections, and 5.3% as severe infections. Table 2 shows the ulcer characteristics.

### 3.3. Microbiological analysis

Based on microbiological laboratory analysis, 89.5% of DFUs showed bacterial growth and were classified as culture-positive, while 10.5% were categorized as culture-negative. Interestingly, although 39.2% of DFUs were not clinically infected, 88.1% of these ulcers (59 out of 67) exhibited bacterial growth. Among culture-positive cases, Gram-positive bacteria (GPB) were significantly more prevalent than Gram-negative bacteria (GNB; 55.6% vs 6.4%), with 27.5% of cultures showing a combination of Gram-positive and Gram-negative bacterial growth. Furthermore, an almost equal distribution of monomicrobial (50.3%) and polymicrobial (49.7%) cultures was observed.

From the 171 diabetic participants with foot ulcers, a total of 278 bacterial isolates were obtained, encompassing 26 bacterial genera and species. Of these isolates, 260 were classified as culture-positive, while 18 were culture-negative. Gram-positive bacteria comprised 65.1% (181/278) of the isolates, GNB accounted for 28.4% (79/278), and 6.5% (18/278) showed no bacterial growth (Table 3).

**Table 3:** Proportion of bacterial isolates from patients with diabetic foot ulcers (N = 260 culture-positive samples).

Bacteria type	N	% (whole)	% from Gram type
GPB	181	65.1	100
MSSA	23	8.3	12.7
MRSA	49	17.6	27
*Enterococcus* spp.	26	9.4	14.4
CoNS	64	23	35.4
*Streptococcus* spp. group B	14	5.0	7.7
*Enterococcus* spp. group D	2	0.7	1.1
Streptococcus spp. group A	3	1.1	1.7
GNB	79	28.4	100
*Pseudomonas* spp.	3	1	3.8
*Proteus* spp.	4	1.4	5
*Alcaligenes* spp.	9	3.2	11.3
*Acinetobacter* spp.	10	3.6	12.7
*Enterobacter* spp.	14	5.0	17.7
*Escherichia coli*	10	3.6	12.7
*Klebsiella* spp.	6	2.2	7.6
*Citrobacter* spp.	2	0.7	2.5
*Haemophilus* spp.	1	0.4	1.3
*Pseudomonas aeruginosa*	2	0.7	2.5
*Klebsiella oxytoca*	3	1	3.8
*Citrobacter koseri*	1	0.4	1.3
*Alcaligenes xylosoxidans*	1	0.4	1.3
*Shewanella*	2	0.7	2.5
*Morganella morganii*	7	2.5	8.8
*Proteus vulgaris* group 3	1	0.4	1.3
*Comamonas* spp.	1	0.4	1.3
*Shewanella putrefaciens*	1	0.4	1.3
*Myroides odoratus*	1	0.4	1.3

CoNS = coagulase-negative staphylococci, GNB = Gram-negative bacteria, GPB = Gram-positive bacteria, MRSA = methicillin-resistant *Staphylococcus aureus*, MSSA = methicillin-sensitive *Staphylococcus aureus*.

Among the 181 Gram-positive isolates, *S aureus* was the most predominant species, representing 39.8% (N = 72) of GPB and 25.9% (72/278) of all isolates. Of the *S aureus* isolates, 68.1% (N = 49) were identified as MRSA. Other GPB included 64 CoNS, 26 *Enterococcus* species, and 17 *Streptococcus* species.

Regarding Gram-negative isolates, the most commonly identified bacteria were *Enterobacter* species (N = 14), followed by *E coli* and *Acinetobacter* species (N = 10 each), and *Alcaligenes* species (N = 9). The detailed proportions of bacterial isolates are summarized in Table 3.

### 3.4. The antibiotic susceptibility patterns

The antibiotic susceptibility patterns for various bacterial isolates are detailed in Table 4. Methicillin-resistant *Staphylococcus aureus* exhibited 100% susceptibility to Tigecycline, Vancomycin, Teicoplanin, and Linezolid, along with high susceptibility to Chloramphenicol. However, it was 100% resistant to Ampicillin. Methicillin-sensitive *Staphylococcus aureus* (MSSA) demonstrated complete susceptibility to multiple antibiotics but was resistant to Ampicillin.

**Table 4:** The antimicrobial susceptibility pattern of Gram-positive and Gram-negative bacterial isolates from patients with diabetic foot ulcers.

Antibiotics tested against GPB organisms		Bacteria						*Enterococcus* group D (n = 2)
		CoNS (n = 64)	MRSA (n = 49)	*Enterococcus* spp. (n = 26)	MSSA (n = 23)	*Streptococcus* group B (n = 14)	*Streptococcus* group A (n = 3)	
		N (%)	N (%)	N (%)	N (%)	N (%)	N (%)	N (%)
Penicillins	Ampicillin	2 (3.1)	0	26 (100)	0	14 (100)	3 (100)	2 (100)
	Oxacillin	24 (37.5)	0	0	23 (100)	0	0	0
Third generation cephalosporins	Ceftriaxone	NT	NT	NT	NT	14 (100)	3 (100)	NT
Fluoroquinolones	Ciprofloxacin	29 (45.3)	28 (57.1)	6 (23.1)	10 (43.5)	NT	NT	1 (50)
	Levofloxacin	32 (50)	29 (59.2)	13 (50)	11 (47.8)	9 (64.3)	3 (100)	2 (100)
Aminoglycosides	Gentamicin	46 (71.9)	40 (81.6)	0	21 (91.3)	0	0	0
Tetracyclines	Tigecycline	64 (100)	49 (100)	26 (100)	23 (100)	14 (100)	3 (100)	2 (100)
Macrolides	Erythromycin	8 (12.5)	14 (28.6)	4 (15.4)	11 (47.8)	2 (14.3)	1 (33.3)	0
Lincosamides	Clindamycin	14 (21.9)	17 (34.7)	0	14 (60.9)	2 (14.3)	3 (100)	0
Glycopeptides	Vancomycin	64 (100)	49 (100)	26 (100)	23 (100)	14 (100)	3 (100)	2 (100)
	Teicoplanin	64 (100)	49 (100)	26 (100)	23 (100)	NT	NT	2 (100)
Oxazolidinones	Linezolid	64 (100)	49 (100)	26 (100)	23 (100)	14 (100)	3 (100)	2 (100)
Miscellaneous protein synthesis inhibitor	Chloramphenicol	60 (93.8)	44 (89.8)	19 (73.1)	23 (100)	14 (100)	3 (100)	2 (100)

Antibiotics tested against GNB organisms		*Enterobacter* spp. (n = 14)	*Acinetobacter* spp. (n = 10)	*E coli* (n = 10)	*Alcaligenes* (n = 10)	*Klebsiella* spp. (n = 9)	*Morganella morganii* (n = 7)	*Proteus* spp. (n = 5)
		N (%)	N (%)	N (%)	N (%)	N (%)	N (%)	N (%)
Cephamycins	Cefoxitin	3 (21.4)	NT	7 (70)	NT	8 (88.9)	5 (71.4)	4 (80)
Third generation cephalosporins	Ceftriaxone	11 (78.6)	1 (10)	2 (20)	NT	4 (44.4)	7 (100)	3 (60)
	Cefotaxime	11 (78.6)	1 (10)	2 (20)	NT	4 (44.4)	7 (100)	3 (60)
	Ceftazidime	12 (85.7)	3 (30)	3 (30)	10 (100)	5 (55.6)	7 (100)	5 (100)
Fourth generation cephalosporins	Cefepime	13 (92.9)	5 (50)	4 (40)	9 (90)	5 (55.6)	7 (100)	3 (60)
Cephalosporins/β-lactamase inhibitors	Piperacillin/ Tazobactam	12 (85.7)	5 (50)	6 (60)	10 (100)	6 (66.7)	7 (100)	5 (100)
Carbapenems	Imipenem	13 (92.9)	5 (50)	10 (100)	10 (100)	8 (88.9)	4 (57.1)	5 (100)
	Meropenem	13 (92.9)	5 (50)	10 (100)	10 (100)	8 (88.9)	7 (100)	5 (100)
	Ertapenem	13 (92.9)	NT	10 (100)	NT	8 (88.9)	7 (100)	5 (100)
Quinolones	Ciprofloxacin	8 (57.1)	4 (40)	1 (10)	3 (30)	5 (55.6)	1 (14.3)	1 (20)
	Levofloxacin	10 (71.4)	4 (40)	2 (20)	5 (50)	6 (66.7)	3 (42.9)	2 (40)
Monobactams	Aztreonam	12 (85.7)	0	3 (30)	1 (10)	4 (44.4)	7 (100)	5 (100)
Aminoglycosides	Gentamicin	13 (92.9)	6 (60)	8 (80)	10 (100)	7 (77.8)	6 (85.7)	3 (60)
	Amikacin	13 (92.9)	5 (50)	10 (100)	9 (90)	8 (88.9)	7 (100)	5 (100)
Tetracyclines	Tigecycline	14 (100)	1 (10)	10 (100)	NT	9 (100)	0	0

Antibiotics tested against GNB organisms		*Pseudomonas* spp. (n = 5)	*Citrobacter* spp. (n = 3)		*Shewanella* spp. (n = 3)	*Myroides odoratus* (n = 1)	*Haemophillus* (n = 1)	*Comamonas* spp. (n = 1)
		N (%)	N (%)		N (%)	N (%)	N (%)	N (%)
Cephamycins	Cefoxitin	NT	2 (66.7)		NT	NT	NT	NT
Second generation cephalosporins	Cefuroxime	NT	NT		NT	NT	1 (100)	NT
Third generation cephalosporins	Ceftriaxone	NT	2 (66.7)		NT	NT	NT	NT
	Cefotaxime	NT	2 (66.7)		NT	NT	NT	NT
	Ceftazidime	4 (80)	2 (66.7)		3 (100)	0	1 (100)	1 (100)
Fourth generation cephalosporins	Cefepime	4 (80)	3 (100)		3 (100)	1 (100)	NT	1 (100)
Cephalosporins/β-lactamase inhibitors	Piperacillin/ Tazobactam	4 (80)	3 (100)		3 (100)	0	1 (100)	1 (100)
Carbapenems	Imipenem	3 (60)	3 (100)		2 (66.7)	1 (100)	1 (100)	1 (100)
	Meropenem	4 (80)	3 (100)		3 (100)	1 (100)	NT	1 (100)
	Ertapenem	NT	3 (100)		NT	NT	NT	NT
Quinolones	Ciprofloxacin	4 (80)	3 (100)		3 (100)	0	NT	0
	Levofloxacin	3 (60)	3 (100)		3 (100)	0	1 (100)	0
Monobactams	Aztreonam	3 (60)	2 (66.7)		2 (66.7)	0	1 (100)	0
Aminoglycosides	Gentamicin	4 (80)	3 (100)		3 (100)	0	NT	1 (100)
	Amikacin	4 (80)	3 (100)		3 (100)	0	NT	1 (100)
Tetracyclines	Tigecycline	NT	3 (100)		NT	NT	NT	NT
Penicillins	Ampicillin	NT	NT		NT	NT	1 (100)	NT
Miscellaneous protein synthesis inhibitor	Chloramphenicol	NT	NT		NT	NT	1 (100)	NT

CoNS = coagulase-negative staphylococci, GNB = Gram-negative bacteria, GPB = Gram-positive bacteria, MRSA = methicillin-resistant *Staphylococcus aureus*, MSSA = methicillin-sensitive *Staphylococcus aureus*, NT = not taken.

Enterococcus species, including *Enterococcus* group D, showed general susceptibility to several antibiotics but exhibited resistance to Oxacillin, Gentamicin, Erythromycin, and Clindamycin. Similarly, *Streptococcus* species were highly susceptible to various antibiotics, although they were resistant to Oxacillin and Gentamicin.

Among GNB, *Enterobacter*, *E coli*, and *Klebsiella* species demonstrated high susceptibility to Tigecycline. In particular, *E coli* showed strong susceptibility to Carbapenems and Amikacin. *Morganella* species were highly susceptible to several antibiotics but resistant to Tigecycline. *Proteus* species also displayed high susceptibility to multiple antibiotics but were resistant to Tigecycline.

*Pseudomonas* species exhibited an 80% susceptibility rate to certain antibiotics, while *Acinetobacter* species showed the highest susceptibility rate (60%) to Gentamicin and were resistant to Aztreonam.

Of the 171 participants, 18.1% (31/171) were identified with MDROs. Among the 49 MRSA isolates, 38.8% (19/49) were classified as multidrug-resistant GPB. Most *Enterococcus* spp. isolates demonstrated resistance to 3 agents across 2 categories: ciprofloxacin, levofloxacin, and gentamicin. The remaining *Enterococcus* spp. isolates were resistant to 1 or 2 of these agents, with no multidrug resistance detected in this group.

Regarding GNB, multidrug resistance was identified in 8 out of 10 *E coli*, 3 out of 9 *Klebsiella* species, and 2 out of 7 *Morganella morganii* isolates. Notably, 4 out of 10 *Acinetobacter* spp. isolates were classified as pandrug-resistant, while 1 out of 10 was extensively drug-resistant.

Management of GPB showed the highest efficacy with Tigecycline, Vancomycin, Linezolid, and Teicoplanin, all exhibiting 100% susceptibility against the tested strains. For GNB, Ertapenem, Meropenem, Amikacin, Imipenem, and Gentamicin were effective, with susceptibility rates of 95.8%, 89.7%, 87.2%, 83.5%, and 82.1%, respectively.

## 4. Discussion

In this study, GPB were the predominant isolates from diabetic patients with foot ulcers, with *S aureus* being the most frequently isolated bacterium, while *Enterobacter* spp. was the most common among Gram-negative bacilli (GNB). The bacteriological profile of DFIs varies depending on disease severity and geographical location. Studies from temperate regions, such as North America and Europe, consistently indicate that aerobic GPB, particularly *S aureus*, coagulase-negative *Staphylococcus*, and *Streptococcus*, are the most prevalent pathogens in DFIs. In contrast, recent studies from tropical and subtropical regions, particularly Asia and northern Africa, have shown that GNBs are the primary pathogens in DFIs, either independently or coexisting with Gram-positive cocci.^[3]^ Notably, the bacterial profile in Southeast Asia differs significantly from that of developed countries, where GPBs predominate.^[12]^

This study found nearly equal proportions of monomicrobial (50.3%) and polymicrobial (49.7%) cultures in DFI samples. Similarly, Sekhar et al reported comparable rates of polymicrobial and monomicrobial infections (44.4%).^[25]^ However, other studies have reported varying findings, with some indicating higher frequencies of monomicrobial infections^[13,26]^ and others showing a predominance of polymicrobial infections.^[4,27]^ These variations likely result from differences in processing techniques and sampling methods, such as the greater sensitivity of tissue cultures in detecting pathogens compared to swab cultures, as well as the chronicity of ulcers.^[26,28]^

The present study found that GPB accounted for 65.1% of isolates, with *S aureus* being the most prevalent, followed by coagulase-negative *Staphylococcus aureus* (CoNS) and *Enterococcus*. Gram-negative bacteria constituted 28.4% of isolates, with *Enterobacter* spp. being the most common, followed by *E coli* and *Acinetobacter* spp. These findings align with previous studies by Macdonald et al, Perim et al, and Mendes et al, which also reported a higher prevalence of GPB in DFIs.^[2,13,29]^

In contrast, studies by Darwis et al and Ismail et al identified a predominance of GNB.^[30,31]^ Research by Du et al and Macdonald et al revealed that GNBs were more prevalent in Upper-Middle and Lower-Middle Income Countries, while GPBs were dominant in High-Income Countries.^[32,33]^ Similarly, studies from Morocco, Lebanon, Egypt, India, and Oman reported a majority of GNB isolates.^[34-38]^ Conversely, Hatipoglu et al observed similar proportions of GPB and GNB over 2 distinct periods.^[14]^

The observed variations in GPB and GNB prevalence across different regions can likely be attributed to environmental factors, hygiene practices, patterns of antibiotic use, and footwear habits.^[8]^

Methicillin-resistant *Staphylococcus aureus* is a significant concern in patients with DFUs, contributing to delayed wound healing, prolonged hospital stays, suboptimal management, and increased healthcare costs.^[9,39]^ In the current study, MRSA accounted for 68.1% of *S aureus* isolates (49/72). This finding aligns with previous studies by Tentolouris et al and Shahrokh et al, which also reported high MRSA prevalence among *S aureus* isolates in DFUs.^[9,40]^ Similarly, Ullah et al found that 76.6% of *S aureus* isolates were MRSA, while Pontes et al and Piri et al reported MRSA proportions of 63.2% and 66.7%, respectively.^[41-43]^ Perim et al observed an even higher percentage of MRSA among *S aureus* isolates (92.6%).^[29]^ Conversely, lower MRSA rates were reported by Akhi et al and Miyan et al, with percentages of 38.2% and 26.7%, respectively.^[39,44]^

In this study, *S aureus* accounted for 25.9% of all isolates, followed by CoNS (23%), *Enterococcus* spp. (9.4%), *Enterobacter* spp. (5%), *Streptococcus* group B (5%), *Acinetobacter* spp. (3.6%), and *E coli* (3.6%). These findings are consistent with global studies showing *S aureus* as the predominant bacterial isolate, with prevalence rates ranging from 17.7% to 32.5%.^[13,29,32,33]^ Similarly, studies from Western and Southern Asia reported *S aureus* prevalence ranging from 19% to 34.6%.^[38,43-45]^ However, other studies identified different predominant bacteria, such as *Pseudomonas* spp., *Proteus mirabilis*, *E coli*, and *Klebsiella pneumoniae*.^[31,36]^

In the present study, GPB, primarily *S aureus*, demonstrated complete susceptibility (100%) to tigecycline, vancomycin, linezolid, and teicoplanin. Among GNB, the most effective antibiotics were ertapenem, meropenem, amikacin, imipenem, gentamicin, and piperacillin/tazobactam, with susceptibility rates of 95.8%, 89.7%, 87.2%, 83.5%, 82.1%, and 79.7%, respectively. Tigecycline and gentamicin were particularly effective against both GPB and GNB, with susceptibility rates of 94.8% and 66%, respectively.

These findings are consistent with numerous studies from various regions reporting the most common bacteria and effective antibiotics (ABs) for treating GPBs and GNBs. For example, this study’s 100% susceptibility of *S aureus* to vancomycin aligns with the results of Piri et al.^[43]^ Similarly, studies by Hatipoglu et al, Ismail et al, and Du et al also identified vancomycin, teicoplanin, and linezolid as highly effective antibiotics against *S aureus*.^[14,31,33]^ Furthermore, Ismail et al reported findings consistent with this study, highlighting the efficacy of imipenem, meropenem, and piperacillin/tazobactam against GNB.^[31]^

Several studies have highlighted the impact of MDROs on DFUs, noting their association with prolonged healing times, increased lower limb amputation rates, and higher mortality.^[46,47]^ However, the variability in definitions and prevalence of MDROs across studies complicates direct comparisons.

In the present study, 38.8% of *S aureus* isolates and 33.3% of Gram-negative Enterobacteriaceae were identified as MDROs. Among the 171 participants, 18.1% had MDROs, 2.3% had pandrug-resistant organisms (PDROs), and 1.8% had extensively drug-resistant organisms (XDROs). Notably, 40% of *Acinetobacter* spp. isolates were PDROs. Due to laboratory limitations, isolates of *Proteus*, *Enterobacter*, *Citrobacter*, and *Pseudomonas* species were excluded, which may explain the relatively low MDRO percentages observed.

Consistent with this study, Hartemann-Heurtier et al and Richard et al reported MDRO prevalence rates of 18% and 23.9% in DFU patients, respectively.^[46,48]^ Similarly, Ismail et al found that 48.3% of isolates were MDROs, with 23.3% classified as XDROs, and 55.1% of GNB isolates being multidrug-resistant.^[31]^ Sannathimmappa et al reported that 38% of Gram-positive pathogens were MDROs, while GNB isolates accounted for 75% of total isolates.^[38]^

In this study, the most prevalent MDRO was MRSA, with 38.8% of *S aureus* isolates being multidrug-resistant. Similarly, Karmakar et al identified MRSA as a significant contributor to multidrug-resistant DFIs.^[49]^ Adeyemo et al found that 80% of DFUs were infected with MDROs, with MRSA accounting for 35.3% of these cases.^[50]^ Studies from Poland and India also reported high MRSA resistance rates, at 70.3% and 55%, respectively.^[51,52]^

This study is one of the few conducted in Jordan that provides valuable insights into the local epidemiology of aerobic bacteria in diabetic patients with foot ulcers. Moreover, the sample size was adequate for the study’s objectives.

This study has several limitations. First, it was conducted at a single-center, which may limit the generalizability of the findings. Second, this study focused exclusively on aerobic bacterial profiles due to the lack of necessary laboratory infrastructure for anaerobic culture testing. While anaerobic bacteria are known to play a role in DFIs, particularly in chronic and necrotic ulcers, the current study’s single-center setting lacked the specialized equipment required for anaerobic isolation and identification. Therefore, the findings presented here primarily reflect the aerobic microbial landscape of DFUs in Jordan.

In conclusion, GPB were the predominant isolates from diabetic patients with foot ulcers, with *S aureus* being the most frequently isolated species. Among GNB, *Enterobacter* spp. was the most commonly identified. The antibiotic susceptibility pattern revealed that vancomycin, linezolid, and teicoplanin were highly effective against GPB, exhibiting 100% susceptibility. Ertapenem, meropenem, amikacin, and imipenem were the most active antibiotics against GNB.

Ongoing surveillance of microbial profiles and antimicrobial susceptibility patterns is essential. Regular studies will help guide empirical therapy and serve as an early alert system for shifts in microbial resistance or susceptibility trends. Additionally, the prescription of antibiotics should be carefully managed, with the implementation or modification of relevant regulations to ensure appropriate use.

## Author contributions

**Conceptualization:** Mohammad Al Gazo, Mohammad El-Khateeb.

**Data curation:** Mohammad Al Gazo, Faris Bakri, Yousef Khader, Laith Baqain, Eid Samara, Jood Aladwan.

**Formal analysis:** Dana Hyassat, Mohammad Al Gazo, Yousef Khader, Eid Samara, Dalia Hanash, Jamal Wadi Al Ramahi, Kamel Ajlouni.

**Investigation:** Yousef Khader, Amro Al-Simadi, Shiraz Mheidat, Dalia Hanash, Mohammad El-Khateeb.

**Methodology:** Mohammad Al Gazo, Yousef Khader, Dalia Hanash, Mohammad El-Khateeb.

**Project administration:** Dana Hyassat, Kamel Ajlouni.

**Resources:** Laith Baqain, Amro Al-Simadi, Shiraz Mheidat.

**Supervision:** Dana Hyassat, Mohammad El-Khateeb.

**Validation:** Mohammad Al Masri.

**Visualization:** Dana Hyassat, Faris Bakri.

**Writing – original draft:** Dana Hyassat.

**Writing – review & editing:** Faris Bakri, Amro Al-Simadi, Mohammad Al Masri, Jood Aladwan, Jamal Wadi Al Ramahi, Kamel Ajlouni.
